# Catch me if you can

**DOI:** 10.7554/eLife.14721

**Published:** 2016-02-26

**Authors:** Emilie Bourdonnay, Thomas Henry

**Affiliations:** 1 CIRI-Centre International de Recherche en Infectiologie, Inserm U1111, Université Lyon 1, Ecole Normale Supérieure, CNRS UMR5308, Lyon, France; 1 CIRI-Centre International de Recherche en Infectiologie, Inserm U1111, Université Lyon 1, Ecole Normale Supérieure, CNRS UMR5308, Lyon, Francethomas.henry@inserm.fr

**Keywords:** trogocytosis, cell to cell spread, francisella tularensis, salmonella typhimurium, Mouse, Other

## Abstract

Direct contact between host cells allows some bacteria to spread within the body without being attacked by the immune system.

**Related research article** Steele S, Radlinski L, Taft-Benz S, Brunton J, Kawula TH. 2016. Trogocytosis-associated cell to cell spread of intracellular bacterial pathogens. *eLife* **5**:e010625. doi: 10.7554/eLife.10625

**Image** Some bacteria exploit a process called trogocytosis to move from cell to cell

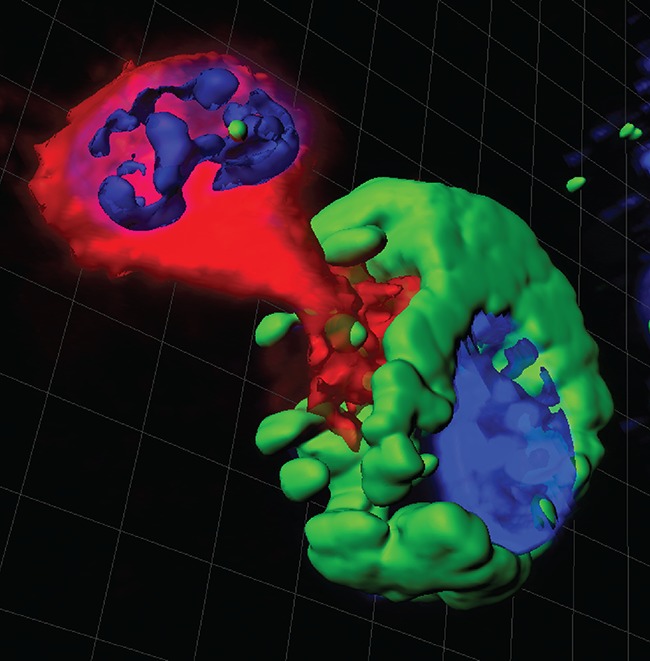

Infectious diseases are one of the main causes of death worldwide. Several of the most deadly bacterial pathogens, such as *Salmonella enterica* and *Listeria monocytogenes*, cause disease by multiplying inside human cells. The human body is constantly patrolled by immune cells, and contains lots of antibodies and other molecules that can target and kill microbes, but the environment inside host cells provides a safe haven where certain pathogens can avoid detection by the immune system. However, each host cell can contain only a limited number of these intracellular bacteria so, after several rounds of replication, they need to leave the infected cell and invade other cells in order to continue multiplying. Until recently the details of how intracellular bacteria can spread between cells without being detected and killed by the immune system were poorly understood.

Now, in eLife, Tom Kawula of the University of North Carolina and colleagues – including Shaun Steele as first author – report how experiments on *Francisella tularensis*, a bacterium that causes a disease known as tularemia, have revealed more details about this process (Steele et al., 2016). *F. tularensis* replicates in the cytosol of immune cells called macrophages and hundreds of them can live inside a single host cell (Celli and Zahrt, 2013). Previous studies have revealed that some intracellular bacteria, including *L. monocytogenes,* grow an actin tail in order to move between epithelial cells (Figure 1; Welch and Way, 2013). However, *F. tularensis* is unable to form an actin tail so it must rely on a different mechanism to spread around the body.

**Figure 1.**
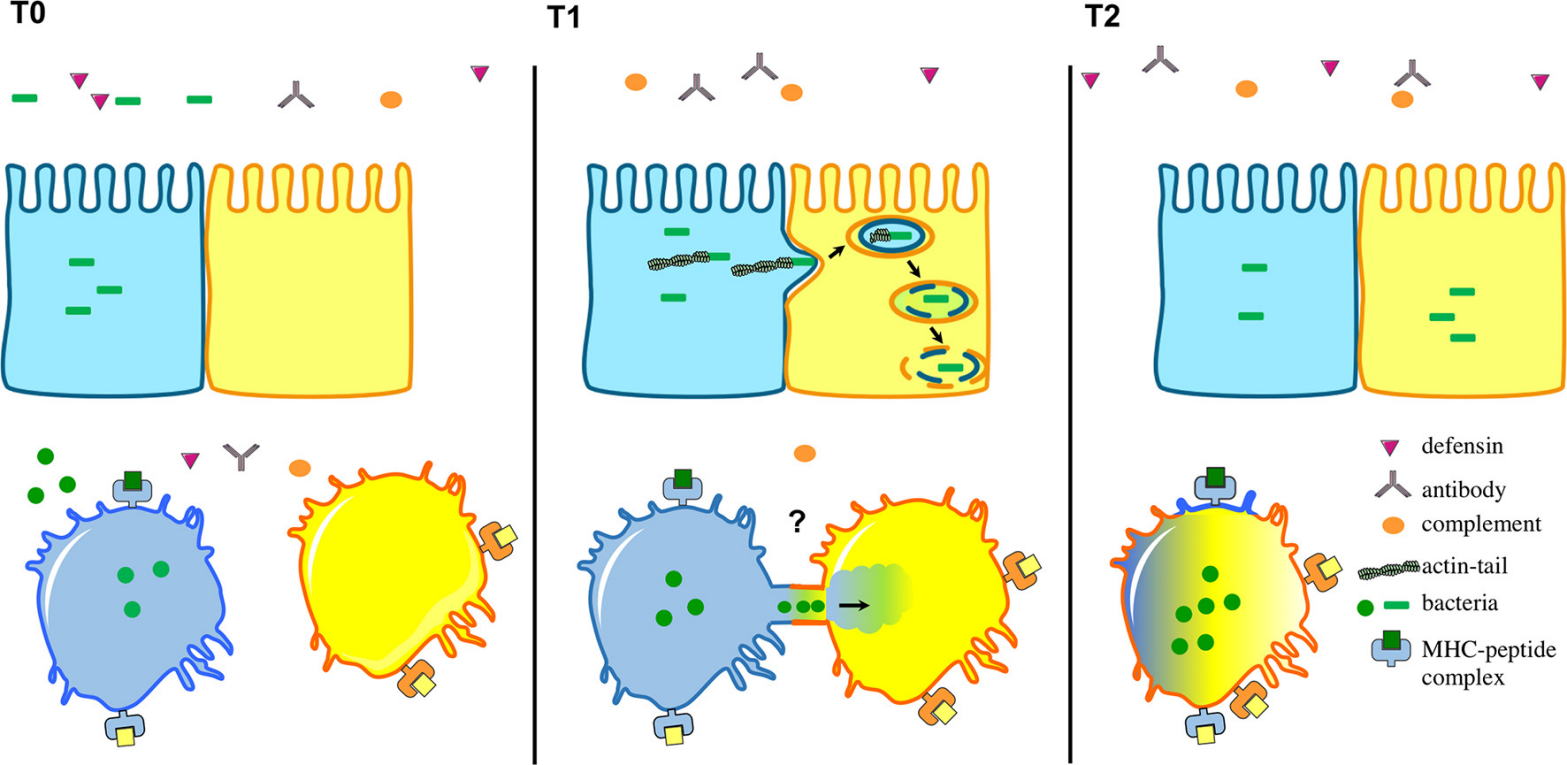
Intracellular bacteria can spread between host cells in different ways. The environment surrounding the host cells is full of immune cells as well as proteins from the complement system (orange), defensins (purple) and antibodies (grey). Top: After several rounds of replication in host cells, certain intracellular bacteria (green), including *L. monocytogenes,* have the ability to make an actin tail that can propel them from an infected cell (shown in blue) towards a neighbouring epithelial cell (yellow). This causes part of the infected cell membrane to bulge into the neighbouring cell, which eventually results in the bacteria being taken into the neighbouring cell surrounded by two layers of membrane (one from each cell). Bacteria then break down the two membranes to be released into the cytosol. Bottom: Steele et al. found that *F. tularensis* and *S. enterica* can transfer between macrophages through a trogocytosis-associated process. Uninfected macrophages (yellow) acquire bacteria from infected macrophages (blue) following temporary contact between the cells. Afterwards, the cells separate and the bacteria start to replicate in the acceptor cell (blue-yellow; the original blue donor cell is not shown). This bacterial cell-to-cell transfer is also associated with the exchange of membrane, membrane proteins and cytosol.

Using video-microscopy, Steele et al. observed that uninfected macrophages acquire *F. tularensis* from infected macrophages following a brief cell-to-cell contact. This process does not kill the cells involved, or expose the bacteria to immune responses outside the cells. Moreover, the exchange of materials between two macrophages is not restricted to pathogens that live in the cytosol: Steele et al. demonstrate that macrophages can also exchange latex beads and plasma membrane. The researchers also demonstrate that *S. enterica –* which lives inside host cell compartments called vacuoles *–* can be transferred between macrophages in the same way. The active exchange of plasma membrane and membrane proteins was first observed in other immune cells called lymphocytes over a decade ago and is called trogocytosis (from the Greek Trogo=to nibble; Joly and Hudrisier, 2003).

The process discovered by Steele et al. joins a list of strategies that bacteria, viruses and other intracellular pathogens are known to use to spread in their host (reviewed in Sattenau, 2008). These other strategies include the fusion of infected cells with surrounding cells, the use of membrane structures that bridge the gaps between cells (Sherer and Mothes, 2008), and the exploitation of the mechanism by which macrophages recognize and engulf dying cells (Czuczman et al., 2014).

Trogocytosis is a normal process that mainly involves macrophages and other immune cells, such as monocytes. It happens more often upon infection, specifically between infected macrophages and their donor cells. This observation suggests that trogocytosis is regulated by individual cells upon infection. A remarkable feature of this process is that membrane proteins that are exchanged between cells – such as the Major Histocompatibility Complex (MHC) molecules, which help to activate immune responses – continue to work properly in their new cell. Therefore, trogocytosis may serve as a surveillance mechanism for the immune system.

The molecular mechanisms underlying trogocytosis and the associated transfer of intracellular bacteria remain to be deciphered. This task will be particularly challenging since it is not currently possible to block trogocytosis with genetic techniques or specific inhibitor drugs. Further experiments are needed to visualize the cellular structures involved in the process, and to understand how the transfer of bacteria is regulated. It will also be important to understand the impact of trogocytosis-associated transfer on other processes, notably innate immune signaling (Ablasser et al., 2013), the presentation of antigens (including the possible transfer of the MHC between cells; Campana et al., 2015), and the spread of the bacteria within the host.
